# The red packet phenomenon from the perspective of young Chinese doctors: a questionnaire study

**DOI:** 10.1186/s12910-022-00793-w

**Published:** 2022-05-30

**Authors:** Hanhui Xu, Mengci Yuan

**Affiliations:** School of Medicine, Nankai University, Tianjin, 300071 China

**Keywords:** Red packets, Medical ethics, Unethical behavior, Chinese young doctors

## Abstract

**Background:**

In China, informal payments in the medical profession, which workers in the public health care system receive from patients in the course of performing profession-related activities, are usually referred to as “red packets” (*Hongbao* 红包). The phenomenon of red packets is widespread and has become one of the most negative factors affecting the doctor-patient relationship in China. Our study aims to explore the situation concerning the phenomenon of red packets in China after the “Red Packet Ban”.

**Methods:**

A questionnaire was developed including general demographic characteristics, asking whether they had ever been offered red packets, whether they had ever accepted red packets, their reasons for accepting the first red packet and so on. We recruited a total of 413 doctors to complete this questionnaire and conducted in-depth telephone interviews with 18 doctors from the initial group.

**Results:**

Our data shows that 73 doctors claimed to have accepted red packets, accounting for 17.7% (73/413) of all respondents and 27.8% (73/263) of doctors who had been provided with red packets. 23.2% of red packets were offered after the operation and 67.1% of the doctors declared that the main reason for accepting the red packet was that they “refused the red packets more than once, but the patients/family members were sincere and it was difficult to refuse.” The total amount of the red packets they received each month accounted for no more than 5% of their income.

**Conclusions:**

(1) The acceptance of red packets does exist among young doctors in China, but shows a significant decrease compared to previous studies. (2) There has been a sharp rise in the proportion of gratitude red packets. (3) Patients should also be educated regarding their behaviour in providing red packets.

**Supplementary Information:**

The online version contains supplementary material available at 10.1186/s12910-022-00793-w.

## Background

Informal payments in the medical profession are generally defined as “payments to individual and institutional providers in-kind or cash that is outside the official payment channels, or are purchases that are meant to be covered by the healthcare system [1].” This is common not only in some developing and transitional countries in Asia, Middle East, Africa and Central and Eastern Europe, but even in some developed countries in Southern Europe like Greece, Italy and Spain [2–6].

In China, informal payments in the medical profession are usually referred to as “red packets” (*Hongbao* 红包), which are officially defined by the health authorities as inappropriate benefits, such as cash, goods, gift cards, and negotiable securities, that workers in the public health care system receive from patients in the course of performing profession-related activities [7]. To facilitate the quantification of statistical data, in the following study, we limit the use of “red packets” to cash, vouchers, and shopping cards provided privately to doctors by patients and their families outside the official payment channels. Consumable goods like fruit, tobacco, alcohol, and cosmetics are not included.

The phenomenon of red packets is widespread [8–12] and has become one of the most negative factors affecting the doctor-patient relationship in China [8, 13–17]. According to previous studies, market-oriented healthcare reform [12, 18–20], low basic income of doctors [19–22], mistrust among patients [10, 11, 22], traditional custom [10, 11, 23, 24], and weak professional ethics of doctor [10] contribute to such phenomenon. Since the 1980s, the Chinese healthcare system has undergone marketisation. This reform pushes public hospitals into the market and causes them to become self-financing by cutting governmental investment. As a result, on the one hand, competition is triggered among hospitals to provide better services for patients. On the other hand, it exacerbates the imbalance between supply and demand. Patients and their families provide informal extra payments to secure quicker or better healthcare services. Secondly, for a long time after the reform, previous guideline prices for consultations and treatment had been retained, which left doctors with a lower basic income. For example, in a big city hospital, the consultation fee of an attending physician, whose monthly salary was 2500 yuan,^1^ was 7 yuan [20]. The low income leads many healthcare practitioners to regard informal payments as an important source of income. Transformation of the health system and low income also contribute to the informal payments phenomenon in some transitional countries, especially in post-communist countries [25]. Thirdly, in China, under the reform, profit-oriented hospitals encourage doctors to prescribe more medication, resulting in over-medication. This worsens the doctor-patient relationship and creates further mistrust among patients. Patients tend to believe that providing red packets is the most direct and effective way to access high-quality medical services. Fourthly, some scholars attributed medical red packets partly to traditional custom. During Qing Dynasty (1636–1912), red packets already existed in medical practice [24]. Before the market-oriented health reform, China had gone through nearly 40 years of a planned economy. Even over this period, red packets were provided by patients to show their gratitude. Lastly, due to the lack of ethical training, some doctors did not see an ethical problem with accepting red packets [10]. They usually follow their own moral judgment and justify the acceptance of red packets by claiming that they deserve it or that it reflects patients’ recognition of their efforts.

Not only does the acceptance of red packets go against the professional ethics of doctors in China, but also against the relevant laws and regulations. *The Law on Licensed Doctors of the People’s Republic of China* states: “Any doctor, who takes advantage of their position to extort and illegally accept patients’ property or seek other illegitimate gains, will be given a disciplinary warning or suspended for a minimum of six months and a maximum of one year. If the circumstances are serious, his or her license will be revoked; If a crime has been constituted, criminal liability will be investigated according to the law [26].” Since the 1990s, Chinese authorities have explicitly classified the acceptance of red packets in the medical profession as corruption. In 1993, the Ministry of Health (MOH) issued a specific ban on red packets [27]. In 2013, the National Health Commission (NHC), the former MOH, issued a policy named as “Promoting medical ethics by prohibiting nine types of behavior in medical practice”, in which accepting red packets is number one on the list [28]. In 2014, NHC issued another policy known as the “Red Packet Ban”, requiring doctors and patients to sign an agreement that they would not make any red packet exchanges [29].

This research contributes to the literature in three regards. Firstly, previous studies on red packets have been more likely to interview the patient, whereas our study has been conducted on the doctor’s side [8, 9, 11, 30]. Secondly, existing studies only reflect the situation prior to 2015, before the phenomenon of red packets had become widespread. In light of the fact that the last five years have received little attention, we seek to determine whether the situation has changed since the “Red Packet Ban” was issued. Thirdly, our study focuses on junior doctors who have not been given particular attention before. Their attitudes and responses to red packets affect not only the doctor-patient relationship in the present but also in the future.

## Methods

### Study population

In this study, we aimed to understand the attitudes and reactions to red packets of young doctors, in surgery-related specialties, who started their careers after the “Red Packet Ban” in 2014, and to investigate whether the acceptance rate of red packets by doctors has decreased in recent years among this group. The geographical area and province of the participants were not restricted. The only criterion for inclusion was that participants must be young Chinese doctors with clinical work experience of no more than 5 years. Snowball sampling was employed to select more participants, and informed consent was obtained.

### Questionnaire development

After an in-depth literature review and internal discussions with investigators who were also doctors, we developed the questionnaire specifically for this study (Additional file 1). The questionnaire consists of three main sections: (1) general demographic characteristics (gender, age, education, title, location, etc.); (2) reactions to being offered a red packet for the first time (job titles when being offered the red packet, nature of the red packet, value of the red packet, reaction, etc.); (3) reason for accepting a red packet for the first time, and perspective of the red packet phenomenon (factors influencing doctors to accept red packets, frequency to be offered red packets in the last year, etc.). In addition, in-depth telephone interviews were conducted with 18 doctors from the participants who completed the questionnaire. All subjects involved in the research were fully informed about the research project and all voluntarily consented to completing the questionnaire and no identifiable information was collected for each individual. The online survey was administered between December 23, 2020, and January 21, 2021 and the survey link was posted via WeChat.

### Statistical analysis

The characteristics of participants were presented as the median and interquartile range (IQR) for continuous variables, and number and percentage for categorical variables. We used Wilcoxon rank-sum test and Pearson’s chi-squared test to describe the differences between doctors who have never been offered red packets and those who have been offered red packets for continuous and categorical variables, respectively. We also reported the number and percentage for each option in the questionnaire. We used Stata 17.0 SE (College station, StataCorp, TX) in all analyses and graphs.

## Results

A total of 413 young doctors were randomly selected for the study, with an average age of 29 years, including 259 males (62.71%) and 154 females (37.29%); 81 (19.6%) with a doctoral degree, 272 (65.9%) with a master’s degree, and 60 (14.5%) with a bachelor’s degree. There were 75 (18.2%) residents, 240 (58.1%) fellows, 90 attending physicians (21.8%) and 8 chief physicians (1.9%). The respondents were from 5 regions, including North-east, Central-South, East, West and North (Table 1). Women accounted for 47.3% of doctors who have never been offered red packets, but only 31.6% of those who have. In terms of age, the median age of those doctors who have never been offered red packets was 2 years younger than those who have. The doctors from the Northeast region including Heilongjiang, Jilin and Liaoning provinces reported a higher percentage (32.3%) to be offered red packets than their proportion (17.3%) of doctors who have never been offered.

**Table 1:** Demographic information

Demographic	N (%) 413	Have never been offered red packets 150	Have been offered red packets 263	*p*-value
*Gender*				
Male	259 (62.7)	79 (52.7%)	180 (68.4%)	
Female	154 (37.3)	71 (47.3%)	83 (31.6%)	**0.001**
Age, median (IQR) years	29 (27–30)	28 (26–30)	30 (28–31)	** < 0.001**
*Education level*				
Bachelor’s degree	60 (14.5)	24 (16.0%)	36 (13.7%)	0.81
Master’s degree	272 (65.9)	97 (64.7%)	175 (66.5%)	
Doctoral degree	81 (19.6)	29 (19.3%)	52 (19.8%)	
*Position*				
Interns	75 (18.2)	48 (32.0%)	27 (10.3%)	** < 0.001**
Resident physician	240 (58.1)	79 (52.7%)	161 (61.2%)	
Attending physician or chief physician	98 (23.7)	23 (15.3%)	75 (28.5%)	
*Location (region)*				
North-east	111 (26.9%)	26 (17.3%)	85 (32.3%)	** < 0.001**
Central-South	86 (20.8%)	26 (17.3%)	60 (22.8%)	
East	75 (18.2%)	28 (18.7%)	47 (17.9%)	
West	19 (4.6%)	14 (9.3%)	5 (1.9%)	
North	122 (29.5%)	56 (37.3%)	66 (25.1%)	

Bold indicates* p* < 0.05 (typically < 0.05) is statistically significant

As shown in Table 1, of the 413 doctors who participated in the questionnaire, 150 (36.3%) said they had never been offered red packets. Thus, the second part of our survey was conducted among the 263 (63.7%) doctors who had been offered red packets. By calculation (Table 2), of these participants, 180 (68.4%) were male and 83 (31.6%) were female, and more than half of them were in their first year at the hospital when they were offered red packets. In terms of providers, the majority of red packets were provided by the patient’s family (83.7%), with only a small proportion (16.3%) being given by the patient themselves. As for the nature of red packets, they were generally offered before the operation (76.0%). When asked about the reaction (Q5, Table 2), 182 participants (69.2%) declared that they refused red packets directly. 63 respondents (24%) did not refuse them directly. Rather, they “returned” such red packets by deducting the value from the patient’s medical bill. Another 4 respondents (1.52%) declared that they handed red packets to the Hospital Supervision Unit. By contrast, only 12 respondents (4.6%) accepted red packets.

**Table 2:** The first time you were offered a red packet

Questions	Responses	N(%)	All
Q1: How long had you worked in hospital when you were offered the first red packet?	1 year	146 (55.5)	263
	2 years	81 (30.8)	
	3 years	22 (8.4)	
	Longer than three years	11 (4.2)	
	Invalid answer	3 (1.1)	
Q2: What was your position when you were offered the first red packet?	Resident	131 (49.8)	263
	Fellow	131 (49.8)	
	Attending and chief physician	1 (0.4)	
Q3: Who provided the red packet?	Patient	43 (16.3)	263
	Patient’s family	220 (83.7)	
Q4: What was the nature of the first red packet?	A pre-surgery red packet	200 (76.0)	263
	An intra-operative red packet	2 (0.8)	
	An after surgery red packet	61 (23.2)	
Q5: What was your reaction when you were offered the first red packet?	Accepted it directly	12 (4.6)	263
	Refused it directly	182 (69.2)	
	Did not refuse it directly, but “returned” it by deducting it from the patient’s medical bill	63 (24)	
	Handed it over to the hospital disciplinary department	4 (1.52)	
	Other	2 (0.76)	
Q6: If you did not accept it directly, what was your reason?	Receiving red packets is against professional ethics	184 (73.3)	251
	The hospital/department banned such activities	32 (12.7)	
	No other doctors accept red packets	2 (0.8)	
	The patient’s condition was too complicated to take responsibility	8 (3.2)	
	The value of the red packet was too large to accept	5 (2)	
	Other______ (please write your reaction)	20 (8)	
Q7: After that, have you since accepted red packets?	No	190 (75.7)	251
	Yes	61 (24.3)	

Of the 251 participants who did not accept the red packets (263 − 12 = 251), when asked why they would not accept them (Q6, Table 2), 184 (73.3%) respondents selected the option of “receiving red packets was against professional ethics”. 32 (12.7%) respondents gave the reason that “the hospital/department did not allow red packets”. 8 (3.2%) respondents chose the option that “the patient’s condition is too complicated to take responsibility”. 5 (2%) respondents declared that “the value of the red packet was too large to accept”. 2 (0.8%) respondents chose the options of “no other doctors accept red packets”. Then, we asked if they had ever received red packets in their career. 61 doctors (42.3%) answered “yes” and 190 (75.7%) doctors answered “never” (Q7, Table 2).

The next part of our survey continued with the 73 doctors (12 + 61) who had previously accepted red packets. As shown in Table 3, among this group, 60 (82.2%) were male and 13 (17.8%) were female. The majority received red packets for the first time (Q3, Table 3) with a value of 500 yuan (58.9%), and 500–1000 yuan (26.0%). 49 (67.1%) of the doctors declared that the main reason for accepting the red packet (Q4, Table 3) was that they “refused the red packets more than once, but the patients/ family members were sincere and it was difficult to refuse”. 11 (15.1%) of the doctors reported that they did so “to give the patient/family peace of mind”. 8 respondents believed that it is fine to keep the red packet because “it was the reward and acknowledgement for hard work”. When asked about their feelings after accepting the red packet for the first time (Q5, Table 3), 40 (54.8%) doctors said that “they felt slightly worried and uncomfortable”, 22 (30.1%) doctors with the feeling of “very uneasy and regretful”. Another 8 (11.0%) doctors said they were “at ease”. In terms of the doctor’s attitude towards the patient after receiving the red packet (Q7, Table 3), 39 respondents (53.4%) said that they would be more patient and warmer to the patient after receiving a red packet without affecting the interests of other patients. However, 8 (11.0%) respondents said that they would give preferential treatment to the patient after receiving a red packet, such as giving priority to the patient in terms of beds or surgery. When asked about the amount and frequency of the red packets they accepted, most of the doctors reported that the largest amount was below 2,000 yuan (Q8, Table 3) since they became doctors, and the frequency was less than once a month (90.4%) over the past year (Q9, Table 3). In addition, the total value of red packets they received each month accounted for no more than 5% (Q10, Table 3).

**Table 3:** For respondents who have ever accepted red packets

Questions	Responses	N (%)	All
Q1: What is your gender?	Female	13 (18.0)	73
	Male	60 (82.0)	
Q2: How long had you worked in the hospital when you accepted the first red packet?	1 year	41 (56.2)	
	2 years	25 (34.2)	
	3 years	4 (5.5)	
	Longer than 3 years	3 (4.1)	
Q3: What was the value of your first red packet?	< 500 yuan (including 500)	43 (58.9)	73
	500–1000 yuan (including 1000)	19 (26.0)	
	1000–2000 yuan (including 2000)	3 (4.1)	
	> 2000 yuan	1 (1.4)	
	Other	7 (9.6)	
Q4: Why did you accept the first red packet?	It was the reward and acknowledgement for my hard work	8 (11.0)	73
	I had refused the red packets more than once, but the patient/ family members were sincere and it was difficult to refuse	49 (67.1)	
	I aimed to give the patient/family peace of mind by accepting their red packet	11 (15.1)	
	Most of my colleagues accept red packets so it seemed fine for me	2 (2.7)	
	Other	3 (4.1)	
Q5: How did you feel after accepting the red packet for the first time?	I felt very uneasy and regretful	22 (30.1)	73
	I felt slightly worried and uncomfortable	40 (54.8)	
	At ease	8 (11.0)	
	Other	3 (4.1)	
Q6: What your attitude to red packets after accepting the first one?	I no longer accept them	19 (26.0)	73
	It was much easier to accept them after the first one	5 (6.8)	
	It depends	49 (67.1)	
Q7: Was there a significant change in your attitude towards a patient after you received a red packet from them?	No significant change	26 (35.6)	73
	I was more patient and warmer, but not at the expense of other patients’ interests	39 (53.4)	
	The patient was given preferential treatment over other patients, for example, by being given priority for a bed or surgery	8 (11.0)	
Q8: What is the value of the largest red packet you have received so far?	< 500 yuan (including 500)	23 (31.5)	73
	500–1000 yuan (including 1000)	24 (32.9)	
	1000–2000 yuan (including 2000)	19 (26.0)	
	2000–5000 yuan (including 5000)	3 (4.1)	
	> 5000 yuan	3 (4.1)	
	Invalid answer	1 (1.4)	
Q9: What was the frequency of accepting red packets over the past year?	Less than once a month	66 (90.4)	73
	1–3 times per month	4 (5.5)	
	4–5 times per month	2 (2.7)	
	More than 5 times per month	1 (1.4)	
Q10: What was the proportion of red packets to your total income last year?	It is unusual and morally unacceptable to accept extra fees from patients	28 (38.4)	73
	It is fine to accept red packets so long as doctors do their best to treat and serve their patients after accepting red packets	36 (49.3)	
	Invalid answers	9 (12.3)	
Q12: What is the reason behind the prevalence of the red packet phenomenon?	The red packet can be regarded as a form of compensation for doctors’ hard work	29 (39.7)	73
	It is the patient’s problem that they felt peace of mind after providing doctors red packets	30 (41.1)	
	There is a “red packet traditional custom” behind such a phenomenon	14 (19.2)	

**Figure1:**
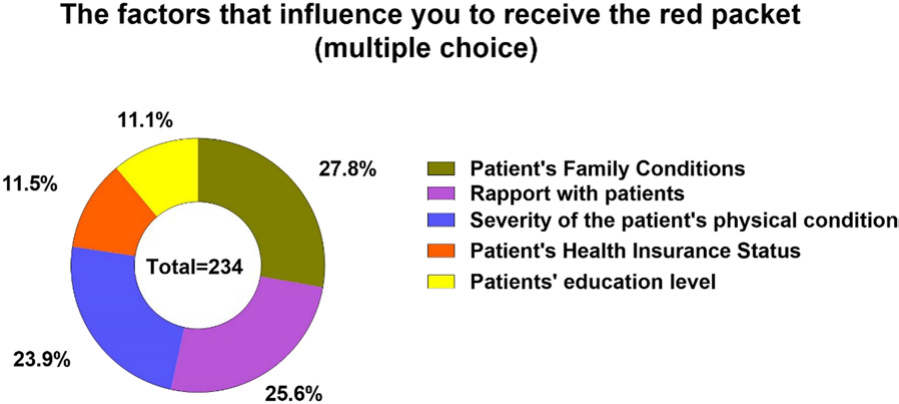
The factors influenced doctors to accept the red packet

Finally, there were a couple of questions related to the doctor’s personal feelings and opinions about the red packet phenomenon. When asked about the factors influencing doctors’ decisions on the acceptance of red packets (Fig. 1), the top three factors were “patient’s financial situations” (89.0%), “the relationship with the patient” (82.2%) and “the complexity of the patient’s disease” (76.7%). In terms of “what do you think about the acceptance of red packets” (Q11, Table 3), 28 respondents (38.4%) claimed that the phenomenon was definitely “unusual” and “it is morally unacceptable to accept extra fees from patients”. By contrast, 36 respondents (49.3%) declared that “it is fine to accept red packets and you should do your best to treat and serve your patients after receiving red packets”. Another 9 respondents provided invalid answers. When asked about the reasons for the prevalence of the phenomenon of red packets, the two main reasons attributed by the respondents were “it is the patient’s problem that they felt the peace of mind after providing doctors red packets” (41.1%) and “the red packet can be regarded as a form of compensation for doctors’ hard work” (39.7%). Another 14 respondents (19.2%) attributed the phenomenon of red packets to “traditional custom” by pointing out that “the custom of red packet exists in many professions”.

## Discussion

The phenomenon of medical red packets has a long history and is widespread in China. This article focuses on the responses and attitudes of young doctors to red packets. The results suggest that, compared with previous studies, some of the findings of our study require more detailed analysis.

Firstly, the acceptance of red packets exists among young doctors in China. Of the respondents, 73 doctors claimed to have accepted red packets, accounting for 17.7% (73/413) of all respondents and 27.8% (73/263) of doctors who had been provided red packets. The true proportion of the doctor community accepting red packets in China may be higher. One reason is that some respondents may hide the truth since such activities are immoral and forbidden. In addition, senior doctors are more likely to be offered red packets.

As previous studies reported, on the doctors’ side, the reasons for accepting red packets are as follows: (1) doctors’ basic income is low in China and red packets are an important source of income for them [19–22]. However, this was not reflected in the present study. When asked how often they received red packets, 90.4% of respondents (66/73) said that they received them less than once a month on average. As for the proportion of red packet income to total income, 89% of doctors (65/73) said less than 5%. This indicates that receiving red packets has little impact on their income. One explanation for the difference between the present study and previous studies is probably that doctors’ incomes have increased over the years. Data shows that in 2020, the average annual salary of doctors in tertiary hospitals was 210,554 yuan [31], which was 2.16 times the national average wage (97,379 yuan) [32]. All respondents were from 3AAA hospitals. Thus, at least for the doctors from 3AAA hospitals, having red packets to compensate for low incomes has declined significantly. By contrast, 35.6% (26/73) of the respondents said that one reason for doctors having red packets was that “the income of doctors who are overworked and burning out in their daily practice does not match their efforts and red packets are considered as a form of compensation”. It seems to indicate that one of the main reasons why doctors accept red packets today is no longer because of their low income, but because their income does not meet their expectations. (2) The practice of informal payments seems to be a social norm in China [10, 11, 23, 24]. In our questionnaire, 19.2% of the respondents (14/73) mentioned this reason. At the same time, it is not easy to refuse red packets offered through social connections [11, 23]. It was also reported by some of the respondents in the study. (3) For some doctors, accepting a red packet is a sign of confidence in their medical abilities and also suggests to the patient or the patient’s family that “if I dare to accept a red packet, I will be able to do the operation well [8, 23].” In our study, one doctor expressed a similar sentiment. He said that if a doctor did not even dare to accept a red packet, he would be perceived by patients as unable to manage the operation.

Secondly, compared to previous survey studies, there was a significant decrease in the acceptance of red packets by doctors. Due to the sensitivity of the issue, although the phenomenon of doctors receiving red packets is mentioned in much of the literature as a common practice, relevant questionnaire surveys targeting the doctor community are not common. A 2002 survey showed that 96.1% of respondents said they had received red packets [33]; another study in 2014 showed 57% [34]. In contrast, only 17.7% of respondents (73/413) in this study said that they had received red packets. Three factors may contribute to this discrepancy that is of concern. First, young doctors have fewer opportunities to receive red packets. In our study, respondents reported that they were offered red packets on average in their second year of clinical entry. Most doctors at this stage do not yet have the authority to operate independently, arrange patients, etc. The red packets for securing quicker or better healthcare services do not seem to be given to these young doctors. Second, as mentioned above, doctors’ incomes have increased over the years and the impact of red packets on their total income has decreased. Third, a series of bans on red packets is working. In 2014, the NHC issued a policy known as the “Red Packet Ban”, requiring doctors and patients to sign an agreement that they would not make any red packets exchanges [29]. It was the first time that the NHC, as the highest medical authority, issued a specific policy on the prohibition of red packets in the twenty-first century. This has been followed by more detailed punitive measures against doctors receiving red packets by provincial and municipal health commissions and hospitals at all levels [35, 36]. Whether these bans work as imagined requires further research.

In contrast to the decline in the proportion of doctors receiving red packets, there has been a significant increase in the proportion of gratitude red packets. In a previous survey of 4000 inpatients from 10 hospitals in China, 54% of respondents said they had given red packets to their doctors. Of these, less than 5% gave red packets out of gratitude [8]. However, in our study, 23.2% of respondents (61/263) reported that patients offered red packets after the operation or when they were about to be discharged from the hospital. Although not all such red packets are motivated by gratitude, considering some patients, especially patients with periodic treatment, may want to be treated better during their next visit. In general, based on previous studies, patients who offered red packets after operation were probably motivated by gratitude [37–39]. One main reason for the significant increase in the proportion of gratitude red packets may be that the respondents in our study were young doctors. As mentioned above, the red packets for securing quicker or better healthcare services do not seem to be given to young doctors. As a result, for young doctors, the proportion of gratitude red packets is much greater. As described in the results, whether participants have been offered red packets varies by gender, age, region, etc. However, the variability analysis is not the purpose of this paper. In addition, gender differences in response to medical red packets have been discussed in another paper [40].

Lastly, our study shows that, on the one hand, the professional ethics of some doctors do need to be further improved; on the other hand, patients should also be educated on their behavior of offering red packets. As mentioned above, in recent years, doctors’ incomes have increased, and the acceptance of red packets to compensate for low incomes has declined significantly. By contrast, some doctors see red packets as a form of compensation for their hard work. In other words, for these doctors, income is not low but still falls short of their expectations, and red packets, though modest, are suitably treated as compensation. This opinion, to some degree, reflects the lack of professional ethics for such doctors. Every profession has its own characteristics, and medical work is accompanied by high pressure and hard work, which should not be a reason to justify the acceptance of red packets. From the perspective of professional ethics, it is a doctor’s duty to do their best to treat patients. Hence, professional ethics education is still necessary.

At the same time, another phenomenon that deserves attention in this study is that patients often offer red packets on their own initiative and do not relent even after being refused. 72.2% of respondents (190/263) said that they had been offered red packets but never accepted them. Of those who had accepted red packets, 67.1% (49/73) reported that they had “refused the red packets more than once but the patients/ family members were sincere and it was difficult to refuse”. This suggests that most doctors did not accept red packets directly when they were offered them by patients. By contrast, they usually rejected the red packets or at least showed that they would not like to accept them.

This seems to be an ethical dilemma for the doctor. On the one hand, rejecting a patient’s red packet may cause psychological frustration and distress to the patient when the patient genuinely believes that the doctor will only provide high-quality health service if the red packet is accepted. As a result, rejecting patients’ red packets may cause the patient to become overly worried. It seems, then, that the doctor should accept red packets out of concern for the interests of their patients, especially as the phenomenon of providing red packets has a cultural basis. On the other hand, accepting red packets is clearly against professional ethics and relevant laws.

The issue can be addressed from two sides. On the doctor’s side, doctors should inform patients that (1) accepting red packets is forbidden and will result in severe punishment for doctors, and (2) the quality of healthcare service will not be compromised by the absence of red packets. If this does not work, then, in order to reduce the negative impact on patients, doctors would be best to not reject red packets directly. Rather, they can accept red packets and then return them by, let’s say, paying the patient’s medical bills. Or they can turn over the money to the Hospital Supervision Unit. These ways to respond to red packets are allowed and even encouraged in many hospitals.

On the patient’s side, they should be informed that red packets will not bring them with extra benefits. That is, the quality of healthcare service will not be impaired by the absence of red packets. It will also cause moral distress to doctors. More importantly, a tougher penalty system for patients providing red packets seems necessary. Current policies and regulations focus on doctors, emphasizing that they are not allowed to accept red packets. However, there seems to be a lack of regulation concerning the practice of providing red packets to doctors from patients. Although, as mentioned above, since 2014, patients have been required to sign an agreement to not offer red packets to their doctors, so far, there are no further measures to restrict such behavior from these patients. Perhaps a more effective way to address the red packet phenomenon would be to prevent patients from offering red packets rather than to unilaterally punish doctors who accept them.

## Limitation

Firstly, the sample size of this study is limited which may cause a bias in results. Secondly, although the participants completed the questionnaire anonymously, and in the knowledge that the contents would only be used for research purposes and would not be disclosed, it is conceivable that some participants may have withheld information due to the sensitivity of the questions. Also, given that younger doctors are less likely to be offered red packets, it is possible that the acceptance of red packets among Chinese doctors in general may be more common. Lastly, most participants in our study were from 3AAA hospitals in large cities. The situation in primary and secondary hospitals might be different considering these hospitals are usually in small and medium-sized cities or towns where the medical resources are scarcer. In addition, the management model of such hospitals may also differ from 3AAA ones. All these factors can actually cause our results to deviate from reality. Therefore, in order to reflect the whole picture of the red packet phenomenon, we intend to conduct further research to expand the sample size of doctors at all levels of hospitals (Additional file 1, 2).

## Conclusion

This study focused on young doctors’ behavior of accepting red packets in China. Compared with previous studies, our study was conducted from the doctors’ perspective and had a much larger sample size. Our study shows, firstly, that although the acceptance of red packets exists among the group of young doctors, the proportion of doctors receiving red packets has significantly decreased compared to previous studies. Secondly, the proportion of gratitude red packets was significantly higher than that shown in previous studies. However, the acceptance of red packets has not completely disappeared among the Chinese medical community. As analyzed above, enhancing professional ethics training for doctors remains an important measure of addressing the issue of red packets and then rebuilding trust between doctors and patients. In addition, patients should also be educated in order to reduce patient-initiated red packet giving. This study provides a basis for understanding the current situation of the doctor-patient relationship in China and offers targeted ideas and suggestions to address the issue of red packets and improve the doctor-patient relationship.

## Supplementary Information

**Additional file 1.** Questionnaire.

**Additional file 2.** Inform consent note.

## Data Availability

The datasets generated and/or analysed during the current study are not publicly available because the raw data includes personal information and accepting red packets is an issue that violates medical ethics and even related laws. Thus, in order to protect the participant’s privacy, the datasets are not publicly available but are available from the corresponding author on reasonable request.

## Footnotes

1 6.38 yuan ≈ 1 USD.
