# Supplementary material for: The red packet phenomenon from the perspective of young Chinese doctors: a questionnaire study
Source: BMC Med Ethics. 2022 May 30;23:56. doi: 10.1186/s12910-022-00793-w (PMC9153131; doi:10.1186/s12910-022-00793-w)
Supplement: Supplementary file 1 — Additional file 1. Questionnaire. [file 12910_2022_793_MOESM1_ESM.docx]

**Questionnaire**

**Informed Consent**

You are cordially invited to take part in a study about the phenomenon of “red packets” within the Chinese medical profession. This research is being conducted by members of the Medical Humanities Research Team at Nankai University, whom seek to determine the attitudes and practices of Chinese doctors receiving red packets. Thank you for agreeing to participate in our research by completing our questionnaire.

Please read and answer the questions carefully. There are no correct answers to the questions, simply choose the most appropriate option.

This questionnaire is entirely anonymous and will not violate your privacy. If you have any questions about the content of the survey, please do not hesitate to contact us.

Yours faithfully,
Nankai University Medical Humanities Research Team

**Red Packets：**To facilitate the quantification of statistical data, we limit the use of “red packets” in the following study to **cash, vouchers, and shopping cards** provided privately to doctors by patients and their families outside the official payment channels. Consumable goods such as tobacco, alcohol, and cosmetics are not included.

**Part I: Basic Information**

**1. Age**

**2. Sex**

A. Male B. Female

**3. Highest Educational qualification**

A. Bachelor's degree B. Master's degree C. Doctorate D. Other

**4. Current position/title**

A. Postgraduate B. Resident C. Attending physician D. Chief physician

**5. The province in which you are currently working**

**6. Have you ever been offered red packets by patients or their families?**

A. Yes (go to Question 7) B. No (end of the questionnaire)

**Part II: The First Time You were Offered a Red Packet**

**7. How long had you been in the hospital when you were offered a red packet for the first time?**

**8. Which province were you residing in when you were offered the first red packet?**

**9. What was the level of the hospital you worked in when you were offered the first red packet?**

**10. What was the department you worked in when you were offered the first red packet?**

**11. What was the provider’s status?**

A. Patient B. Patient's family

**12. What was your status when you were offered the first red packet?**

A. Postgraduate B. Resident C. Attending physician D. Chief physician

**13. What was the nature of your first red packet?**

A. A pre-surgery red packet

B. An intra-operative red packet

C. An after surgery red packet

**14. How did you react to the red packet?**

A. Accepted it directly (go to Question 21)

B. Refused it directly

C. Did not refuse it directly, but “returned” it by subtracting that amount from the patient's medical bill

D. Handed it over to the hospital disciplinary department

E. Other (please write down your reaction)

**15. If you did not accept it directly, was your reason because**

A. Receiving red packets goes against professional ethics

B. The hospital/department banned such pratices

C. The patient's condition was too complicated to take responsibility

D. The content of the red packet was too large to accept

E. No other doctors accepted the red packets in my department/hospital

F. Other (please write down your reaction)

**16. Have you ever accepted red packets after that?**

A. No (end of the questionnaire)

B. Yes (go to Question 17)

**Part III: The First Time You Accepted a Red Packet**

**17. How long had you been in that hospital when you accepted a red packet for the first time?**

**18. Which province were you residing in when you accepted the first red packet?**

**19. What was the level of the hospital you worked in when you accepted the first red packet?**

**20. What was the department you worked in when you accepted the first red packet?**

**21. Why did you accept the red packet at that time?**

A. It was the reward and acknowledgement for my hard work

B. I had refused the red packet more than once, but the patient/ family was sincere and it was difficult to refuse

C. To give the patient/family peace of mind

D. Most of my colleagues accepted red packets, which made me think that it would be fine.

E. Other________.

**22. How much was the red packet?**

A. Less than 500 yuan (including 500 yuan)

B. 500-1000 yuan (including 1000 yuan)

C.1000-2000 yuan (including 2000 yuan)

D. 2000-5000 yuan (including 5000 yuan)

E. Above 5,000 yuan

**23. Your feeling after accepting that red packet**

A. Very uneasy and regretful

B. Slightly worried and uncomfortable

C. At ease

D. Other (please write down your feelings)

**Part IV: Your Attitude Towards Red Packets as a Physician**

**24. What is your feeling and attitude to red packets after accepting the first one**

A. I no longer accept them

B. They were much easier to accept after the first one

C. It depends on the situation

**25.(This is a multiple-choice question) What are the factors that influence you to accept the red packet?**

A. The complexity of the patient’s disease

B. The patient’s financial status

C. Whether the patient has medical insurance or not

D. The patient's education level

E. The patient-physician relationship

**26. Was there a significant change in your attitude towards a patient after you received a red packet from that patient?**

A. I became more patient and warmer, but not at the expense of other patients’ interests

B. The patient was given priority over other patients, for example, being given priority for a bed or surgery

C. No significant change

**27. Was there a significant change in the patient’s attitude towards you after you received a red packet from him or her?**

A. No significant change

B. Yes (Please write down the details)

**28. The largest red packet you have received so far**

A. Below 500 yuan (including 500 yuan)

B. 500-1000 yuan (including 1000 yuan)

C. 1000-2000 yuan (including 2000 yuan)

D. 2000-5000 yuan (including 5000 yuan)

E. Above 5000 yuan

**29. What value of red packet would make you reluctant to accept?**

**30. The frequency of being offered red packets over the past year**

A. Less than once a month

B. 1-3 times per month

C. 4-5 times per month

D. more than 5 times one month

**31. The frequency of accepting red packets over the past year**

A. Less than once a month

B. 1-3 times per month

C. 4-5 times per month

D. More than 5 times one month

**32. In proportion to your total income, how much did you receive in red packets last year?**

A. Less than 5%

B. 5%-10%

C.10%-20%

D. More than 20%

**33. What do you think about the acceptance of red packets?**

A. It is unusual and morally unacceptable to accept extra fees from patients

B. It is fine to accept red packets, so long as doctors do their best to treat and serve their patients after a red packet has been accepted

C. Other

**34.** **The reasons behind the prevalence of the red packet phenomenon**

A. The patient feels peace of mind after providing doctors with red packets

B. The decline of ethics among doctors

C. Inadequate hospital supervision

D. The red packet can be regarded as a form of compensation for doctors’ hard work

E. There is a “red packet traditional custom” behind such a phenomenon

F. Other (Please write down your views)
