# Supplementary material for: The red packet phenomenon from the perspective of young Chinese doctors: a questionnaire study
Source: BMC Med Ethics. 2022 May 30;23:56. doi: 10.1186/s12910-022-00793-w (PMC9153131; doi:10.1186/s12910-022-00793-w)
Supplement: Supplementary file 2 — Additional file 2. Inform consent note. [file 12910_2022_793_MOESM2_ESM.docx]

**Informed Consent**

You are cordially invited to take part in a study about the phenomenon of “red packets” within the Chinese medical profession. This research is being conducted by members of the Medical Humanities Research Team at Nankai University, whom seek to determine the attitudes and practices of Chinese doctors receiving red packets. Thank you for agreeing to participate in our research by completing our questionnaire.

Please read and answer the questions carefully. There are no correct answers to the questions, simply choose the most appropriate option.

This questionnaire is entirely anonymous and will not violate your privacy. If you have any questions about the content of the survey, please do not hesitate to contact us.

Yours faithfully,
Nankai University Medical Humanities Research Team
